# Distributed Ledger Technology in genomics: a call for Europe

**DOI:** 10.1038/s41431-019-0512-4

**Published:** 2019-09-16

**Authors:** Scott Thiebes, Matthias Schlesner, Benedikt Brors, Ali Sunyaev

**Affiliations:** 1 Department of Economics and Management, Karlsruhe Institute of Technology, Kaiserstr. 89, 76133 Karlsruhe, Germany; 2 Bioinformatics and Omics Data Analytics, German Cancer Research Center (DKFZ), Im Neuenheimer Feld 280, 69120 Heidelberg, Germany; 3 Division of Applied Bioinformatics, German Cancer Research Center (DKFZ), Im Neuenheimer Feld 280, 69120 Heidelberg, Germany

**Keywords:** Data publication and archiving, Data processing

Modern life sciences with their highly sensitive omics data face several challenges regarding data storage and sharing [1, 2]. On the one hand data must be protected to preserve the privacy of those individuals who contributed their data to research. On the other hand, omics data’s true value is only to be realized if shared with as many researchers as possible. In an ideal world, patients can flexibly control access to their personal data on a case-by-case basis [3]. However, granting and revoking access to data is a slow and tedious process within the current life sciences research paradigm, where most data is either stored on central controlled-access data repositories or kept locally within the respective research groups [3].

Distributed Ledger Technology (DLT; e.g., Blockchain) recently emerged as a means to enable immutable transactions between untrustworthy parties, which are kept in a consistent state through automated, algorithm-based consensus building mechanisms, thus eliminating the need for third-party trust enforcement and giving way for patients’ direct control over the flow of their personal data [3, 4]. Furthermore, as a distributed database, DLT provides enhanced data availability and integrity compared with centralized data repositories [4, 5]. However, DLT is a novel technology and although it has attracted tremendous attention from practitioners and researchers, the genomics community only recently started to realize DLT’s potential for the field [3, 6]. Overall, we still lack a profound understanding of the benefits that DLT’s application in genomics may yield as well as associated challenges, which is why we want to draw fellow researchers’ attention to some of the most important opportunities and most pressing challenges for DLT in genomics. We think that DLT’s application in genomics can especially bring forth the following opportunities.

1. Providing patients with flexible and direct access over the flow of their personal (genome) data, thus stimulating greater willingness to contribute data to research.
2. Increased security for genome data storage through decentralization and elimination of single points of failure.
3. Although initiatives like ELIXIR already seek to facilitate data sharing in the life sciences, DLT’s inherent characteristics can strengthen such efforts, facilitating further democratization of (genome) data access and the breaking up of extant data silos.

At the same time, we see the following main challenges for DLT’s meaningful application in genomics:

1. DLT was not designed to handle omics-sized data sets.
2. Diverse and uncertain regulatory environments around DLT (e.g., putting genomic data on a distributed ledger is a difficult-to-reverse decision, which might require very high efforts and thus contradict the right to be forgotten as stipulated by the European Union’s General Data Protection Regulation (GDPR)).
3. Applications of DLT in genomics currently mainly revolve around young businesses like Nebula Genomics, EncrypGen, or Genecoin, to name but a few, that aim at building marketplaces where consumers can trade their private genome data for tokens. While such monetization of genome data sharing will likely attract an increasing number of people to share their genome data, many will do so predominantly for monetary reasons without being fully aware of the implications, thus creating new ethical challenges.

European researchers have traditionally been leading voices about the dangers and ethical implications of large-scale access to and use of genome data [7]. Genomics is highly regulated within Europe and genome tests for medical or predictive purposes must be carried out by trained professionals in most European Union member states [8]. Consequently, compared with the US and other regions of the world, the direct-to-consumer genetic testing market in Europe, for example, is much smaller and there are fewer incentives for building DLT-based genome data markets where European consumers can trade their private genome data. However, while the past has taught us that one can easily fall behind in the fast-paced technology sector, we also firmly believe in the European way of cautiously balancing the benefits of large-scale genome data access with the personal and societal risks that arise with such. Building on the spirit that has spawned initiatives for the open and large-scale sharing of genome data [9], we therefore call for more attention to the surging phenomenon of DLT in genomics from European researchers and institutions. In particular, we see the following avenues for European researchers and institutions to contribute to the proliferation of DLT in genomics with a European character. First, we should focus on applying DLT as a tool for researchers that allows individuals to contribute to genomic research while putting less emphasis on establishing yet another genome data market for consumers. Although researchers have already begun to do so (e.g., Lee et al. [10], Ozercan et al. [3], or the iDASH Privacy and Security Workshops 2018 and 2019), more efforts in this direction are necessary. Second, we should investigate means for how DLT can manage omics-sized data while still providing strong information security and privacy in accordance with contemporary European legislation like the GDPR. Third, create a European distributed ledger for genomics (e.g., a European genomics Blockchain), which serves as a lighthouse project, helps breaking up extant data silos, and encourages collaborations among researchers from different institutes and countries across and beyond Europe. Thereby, extant initiatives like ELIXIR could be a fruitful starting ground for setting up such a European distributed ledger for genomics.

Although only time can tell whether DLT can live up to the current hype and meet everyone’s expectations, we believe that in pursuing these avenues, we will be able to realize DLT’s full potential for genomics, beyond mere genome data markets.
